# Porcine Reproductive and Respiratory Syndrome (PRRS) Epidemiology in an Integrated Pig Company of Northern Italy: A Multilevel Threat Requiring Multilevel Interventions

**DOI:** 10.3390/v13122510

**Published:** 2021-12-14

**Authors:** Giovanni Franzo, Giacomo Barbierato, Patrizia Pesente, Matteo Legnardi, Claudia Maria Tucciarone, Giampietro Sandri, Michele Drigo

**Affiliations:** 1 Department of Animal Medicine, Production and Health (MAPS), University of Padua, 35020 Padova, Italy; giacomo.barbierato@studenti.unipd.it (G.B.); matteo.legnardi@unipd.it (M.L.); claudiamaria.tucciarone@unipd.it (C.M.T.); michele.drigo@unipd.it (M.D.); 2 Laboratorio Tre Valli, 37132 Verona, Italy; patrizia.pesente@aia-spa.it (P.P.); gianpietro.sandri@veronesi.it (G.S.)

**Keywords:** porcine reproductive and respiratory syndrome, molecular epidemiology, ORF7, phylodynamics, phylogeography, Italy, evolution, pig flows

## Abstract

Porcine reproductive and respiratory syndrome (PRRS) is probably the most relevant viral disease affecting pig farming. Despite the remarkable efforts paid in terms of vaccination administration and biosecurity, eradication and long-term control have often been frustrated. Unfortunately, few studies are currently available that objectively link, using a formal statistical approach, viral molecular epidemiology to the risk factors determining the observed scenario. The purpose of the present study is to contribute to filling this knowledge gap taking advantage of the advancements in the field of phylodynamics. Approximately one-thousand ORF7 sequences were obtained from strains collected between 2004 and 2021 from the largest Italian pig company, which implements strict compartmentalization among independent three-sites (i.e., sow herds, nurseries and finishing units) pig flows. The history and dynamics of the viral population and its evolution over time were reconstructed and linked to managerial choices. The viral fluxes within and among independent pig flows were evaluated, and the contribution of other integrated pig companies and rurally risen pigs in mediating such spreading was investigated. Moreover, viral circulation in Northern Italy was reconstructed using a continuous phylogeographic approach, and the impact of several environmental features on PRRSV strain persistence and spreading velocity was assessed. The results demonstrate that PRRSV epidemiology is shaped by a multitude of factors, including pig herd management (e.g., immunization strategy), implementation of strict-independent pig flows, and environmental features (e.g., climate, altitude, pig density, road density, etc.) among the others. Small farms and rurally raised animals also emerged as a potential threat for larger, integrated companies. These pieces of evidence suggest that none of the implemented measures can be considered effective alone, and a multidimensional approach, ranging from individual herd management to collaboration and information sharing among different companies, is mandatory for effective infection control.

## 1. Introduction

Porcine reproductive and respiratory syndrome (PRRS) is caused by two viruses: porcine reproductive and respiratory syndrome virus 1 (PRRSV-1) and porcine reproductive and respiratory syndrome virus 2 (PRRSV-2), which have been recently classified in the species *Betaarterivirus suid* 1 and *Betaarterivirus suid 2*, genus *Betaarterivirus*, family *Arteriviridae* (https://talk.ictvonline.org/taxonomy/, (accessed on 10 November 2021)). The PRRSV genome is a single-stranded, positive-sense RNA (ssRNA (+)) viral genome of ~15 kb [1]. Like other ssRNA (+) viruses, it is featured by a high evolutionary rate, involving both frequent mutations (~10^−3^–10^−5^ substitutions/site/year) and recombination events [2,3,4]. Additionally, deletions involving broad regions of the viral genome have been described, although their biological role and consequences on pathogenesis are not clear [5,6,7]. Approximately three-quarters of the genome is occupied by ORF1a and ORF1b, encoding for 14 non-structural proteins, while the terminal part consists of eight partially overlapping ORFs (ORF2a, ORF2b, ORF3, ORF4, ORF5, ORF5a, ORF6 and ORF7) [1]. These proteins, and especially the Glycoprotein 5 (GP5) and Nucleocapsid (N) (encoded by ORF5 and ORF7), are particularly investigated and sequenced for their biological and epidemiological relevance since they have been traditionally used for strain classification and molecular epidemiology studies [8,9,10], benefitting of their high genetic heterogenicity enhanced by immune-driven selective forces. PRRS is considered one of the most, if not the most, impacting disease affecting pig farming and is responsible for major economic losses [11,12]. PRRSV causes significant production losses due to reproductive failure, including abortions, stillbirth, and premature farrowing. In growing pigs, it can cause respiratory and systemic diseases, growth retardation, decreased productive performances, and mortality. The clinical signs can be particularly severe when associated with co-infecting viruses and bacteria. Additionally, the costs and farm management complications required for its control cannot be underemphasized. Different commercial vaccines and vaccination strategies have been developed and widely applied over time. Nevertheless, due to both genetic and phenotypic variability, affecting the among-strains cross-protection, and viral avoidance and subversion of innate and adaptive immune responses, their efficacy is often sub-optimal [13,14]. The application of effective biosecurity measures is therefore of special relevance, to prevent viral introduction and spreading in an area and/or farm [11]. However, this task has proven extremely challenging since PRRSV efficiently transmitted between farms through direct and indirect contacts, including animal movements, semen, fomites, and airborne transmission [11,13,15]. Despite this evidence, a limited number of studies have thoroughly investigated the determinants affecting virus spreading, and most of the data are ascribable to occasional reports or remain limited to the study of single factors. Additionally, in Italy, traditional epidemiological data are sparse. The prevalence of farms infected by PRRSV has been estimated at around 90%, although not always associated with clinical signs [16]. Genetically, two main clades, both belonging to Type 1 subtype 1 [17], have been identified so far and comprise the vast majority of Italian strains, likely resulting from independent introduction followed by local evolution [10]. However, the determinants of the observed local scenario have never been investigated.

An objective and data-driven approach to PRRS control is still perfectible, and much has been delegated to veterinary experience and intuition, or practical constraints. Recently, Alkhamis et al. (2017) and Makau et al. (2021) [18,19] attempted to link PRRSV genetics to environmental factors potentially affecting its epidemiology in North America. Although this innovative work is of extraordinary relevance and interest, the organization and management of the pig farm system were not considered and integrated within the study. Additionally, the obtained results cannot be a priori extended to other geographical regions and production systems because of their peculiarities, which are likely to severely affect PRRSV between-farm spreading and persistence in an area. The present study aims to further extend the study by Alkhamis et al. (2017) by benefitting from a dataset of almost a thousand sequences collected over a 17 year-period (2004–2021) from the largest pig company operating in Northern Italy (hereafter named the *Company*). Particularly, the *Company* is organized according to a strong hierarchical structure, with several (production) pig flows working in a three-site production system, and major efforts are thus paid to preserve the separation among these units, minimizing or preventing direct and indirect contacts among independent production flows. The interplay among PRRSV evolution and epidemiology, the features of the considered area (i.e., geographic and climatic features, anthropic pressure, animal population, etc.), and the management of pig production systems were considered and analyzed in a single/coherent framework, benefitting of the remarkable advantages that have characterized the field of viral phylodynamics. A broad collection of statistical methods has been applied to the Italian scenario to formally and objectively evaluate the risk factors for PRRSV presence and spread. The consequent identification of potential control points could be of benefit for both field veterinarians and managers, thus improving company administration and therefore animal health and welfare.

## 2. Materials and Methods

### 2.1. Dataset Preparation

Complete ORF7 sequences (387 nucleotide long) were obtained during the diagnostic activity of the *Company* in the period 2004–2021 and originated from samples collected for monitor activity or, more commonly, in presence of clinical outbreaks. PRRSV detection and sequencing were performed as described by Drigo et al. [20]. Most of the sequences were associated with the following metadata: collection date, location (longitude and latitude), integrated production chain, and productive site (i.e., sow herds, nurseries, and finishing units). The initial dataset was refined by selecting only the sequences whose complete metadata and high-quality ORF7 (evaluated thorough chromatogram inspection and assessment of the absence of premature stop codon or out of frame mutations) were available. Selected sequences were aligned to the ones of the vaccines used in Italy, and those clustering with vaccine strains were excluded from further analysis. Sequences were then aligned at codon level using the MAFFT [21] method implemented in TranslatorX [22], and the presence of recombination events was assessed using RDP4 [23] and GARD [24] to remove them from the dataset. In fact, the presence of recombinant strains would severely bias the analysis results since different evolutionary histories would be incorrectly estimated using a single phylogenetic tree. The presence of an adequate phylogenetic signal was assessed using the likelihood mapping approach, implemented in Iq-Tree [25], while the presence and strength of temporal signal were assessed using TempEst [26].

### 2.2. Reconstruction of Viral Population Dynamics and Within-Company Spreading

Viral population parameters (time to the most recent common ancestor (tMRCA), evolutionary rate, population size over time, etc.) were reconstructed using the serial coalescent approach implemented in BEAST 10.1 [27]. The substitution model was selected based on the Bayesian Information Criterion (BIC) calculated using Jmodeltest [28], while the relaxed lognormal molecular clock was preferred over the strict one based on the Bayesian Factor (BF) calculation through marginal likelihood estimation performed using stepping stone (SS) and path sampling (PS) approaches [29]. The viral population dynamics were reconstructed over time using the SkyGrid model [30]. Additionally, the directionality and intensity of virus diffusion among productive stages were assessed using a discrete trait analysis (DTA), selecting an asymmetric model with Bayesian Stochastic Search Variable Selection (BSSVS). The final estimations were obtained by performing a 200 million generation Markov chain Monte Carlo run, sampling parameters and trees every 20,000 generations. Results were inspected using Tracer 1.6 (BEAST package) and accepted if mixing and convergence were adequate and the estimated sample size was greater than 200 for all parameters. Parameter estimation was summarized in terms of mean and 95% highest posterior density (HPD) after the exclusion of a burn-in equal to 20% of the run length. Maximum clade credibility (MCC) trees were constructed and annotated using Treeannotator (BEAST package). Data summary statistics and pictures were generated in R, benefitting from the libraries *ggplot2* [31] and *ggtree* [32].

### 2.3. Strain Migration among Integrated Pig Productive Chains

The organization of the Company is featured by a marked hierarchical organization, with pig flows going unidirectionally from sow farms (Site1) to nurseries (Site2) and finally to finishing units (Site3). More in detail, the multi-site pig-production system consists of 18 different pig-flows originating from 20 different sow-herds (Site 1). Every sow-herd delivers pigs to a specific set of Nursery-sites (Site 2) (the total number of nursery sites exceeds 80), all of them operated an all-in/all-out (AI/AO) approach. Finally, weaned-pigs are moved in over 380 finishing units (Site 3) of different sizes, ranging from 1500 to 10,000 heads, operating AI/AO. Direct and indirect contacts among independent integrated flows are minimized as much as possible. Nevertheless, the effectiveness of such separation is hard to objectively evaluate. For this purpose, PRRSV migration among integrated productive chains of the Company was assessed using a structured coalescent approach. Briefly, according to this model, the considered population was divided into a series of demes (i.e., the integrated productive flows), which can be considered as different islands, featured by their own population size and interconnected by a certain migration rate among them. Considering the high number of potential demes, the BAyesian STructured coalescent Approximation (BASTA) [33] implemented in BEAST2 [34] was selected since it combines the accuracy of previous structured coalescent models [35] with the computational efficiency required to handle several populations. Additionally, farms belonging to integrated productive flows for which few sequences were available were merged into a single category to reduce the computational complexity. Finally, the presence of other pig farms and companies operating in the same Italian area and participating in and mediating the viral transmission among integrated productive chains of the Company was accounted for by adding a “ghost” deme (i.e., a deme for which no sequences were available) [36]. An additional model was also generated assuming the presence of 2 ghost demes, one representing other integrated pig companies and the other the unstructured/single/rural farms). Substitution and clock models were selected as previously described. In both cases, the parameters were estimated performing a 200 million generation Markov chain Monte Carlo run, sampling parameters and trees every 20,000 generations. Obtained posterior estimations were managed as described in Section 2.2.

### 2.4. Continuous Phylogeographic Analysis

The migration of PRRSV over time and space was reconstructed using the continuous phylogeographic approach described by Lemey et al. (2010) [37] and implemented in BEAST 1.10 [27]. The substitution model and molecular clock were selected as previously described. Similarly, the migration model (Lognormal relaxed random walk) was chosen using the marginal likelihood calculation approach. The final estimations were obtained by performing a 200 million generation Markov chain Monte Carlo run, sampling parameters and trees every 20,000 generations. Results were visually inspected using Tracer 1.6 (BEAST package).

The reconstruction of PRRSV movements in Italy was obtained using SpreaD3, summarizing and visualizing the full posterior distribution of trees obtained in continuous phylogeographic analyses.

The obtained posterior tree distribution was also used to estimate and statistically evaluate the impact of different environmental variables (reported in Figure S1) on PRRSV strain distribution and dispersal speed as described by Dellicour et al. (2016) [38] using the *seraphim* R package [39]. Original raster encoding the environmental variables of interest was initially managed using the *raster* package in R.

The distance, duration, and velocity of virus spatial dispersal were extracted as vectors that were used to obtain different summary statistics of viral spreading, including dispersal velocity and maximal wavefront distances (measured from the location of the tree root). Additionally, the following issues were investigated:

1. The viral lineages tendency to remain in and/or to disperse to areas associated with higher raster (i.e., environmental variable) values. For this purpose, two statistics were calculated: E (i.e., the mean of the environmental values extracted at the nodes’ position) and R (i.e., the proportion of branches for which the environmental value recorded at the oldest node position is higher than the environmental value recorded at the youngest node position). Therefore, E measures the tendency of tree nodes to remain located in lower/higher environmental values, while R measures the tendency of lineages to disperse towards lower/higher environmental values. To account for phylogenetic uncertainness, these two statistics were computed from a sample of 100 posterior trees, obtaining their posterior distribution, which was compared to a null distribution of the same metric computed after having randomized phylogenetic node positions within the study area, under the constraint that branch lengths, tree topology, and root position are unchanged. Thus, their statistical significance was assessed by BF calculation. For example, the BF associated with the statistic E was calculated as the posterior odds that E_estimated_ > E_randomised_ (if the environmental variable is considered to attract the lineages, the opposite E_estimated_ < E_randomised_ if repulsing them) divided by the equivalent prior odds (assuming the prior probability for E_estimated_ > E_randomised_ to be 0.5). The same approach was used for the R statistics.
2. The association between a particular environmental variable and the dispersal velocity of the considered strains. Two models of spatial movements were considered: (1) “straight line (SL) path” model, assuming a straight movement between the starting and ending locations of each branch (i.e., the branch weight is computed as the sum of raster cells covered by the straight line); (2) “least cost (LC) path” model, using a least-cost algorithm (i.e., the branch weight is computed as the sum of the values of cells transition values between neighboring cells along the least-cost path). In this model, the analyzed environmental variable can be considered both as a conductance (i.e., favoring viral dispersal through the cells with higher values) or resistance factor (i.e., allowing an easier dispersal through cells with lower values). Both instances were tested for each considered factor. The obtained “environmental” weights were used to calculate a regression with the branch duration, and the corresponding coefficient of determination (R^2^_env_) was obtained. A null coefficient of determination (R^2^_null_) was also calculated assuming a null raster (i.e., assuming that only the spatial distance of each movement affects the branch duration). The statistic Q = R^2^_env_ − R^2^_null_ was selected as the final outcome and describes how much the regression is strengthened when the spatial variation in the environmental variable is included. To account for the phylogenetic uncertainness, the Q statistic was calculated for a set of 100 trees sampled from the posterior distribution. A proportion of more than 90% of positive Q values > 0 was considered suggestive of environmental values effect and further tested through BF calculation. For this purpose, the same randomization procedure previously described was used and the BF for an environmental factor was approximated by the posterior odds that Q_estimated_ > Q_randomised_ divided by the equivalent prior odds (still setting the prior probability for Q_estimated_ > Q_randomised_ to 0.5).

## 3. Results

### 3.1. Dataset

A total of 1008 ORF7 sequences (Acc.Numbers OL698868-OL699876; Figure S2) were collected from several integrated production flows in the period 2004–2021 and merged with reference vaccine sequences. After database refinement, 982 were maintained for further analysis and included in the final dataset. No recombination events were detected in the analyzed region, as previously reported [8,40], and the phylogenetic and temporal signals proved adequate for further analysis.

### 3.2. PRRSV Strain History, Evolution, and Population Dynamics

**Figure 1:**
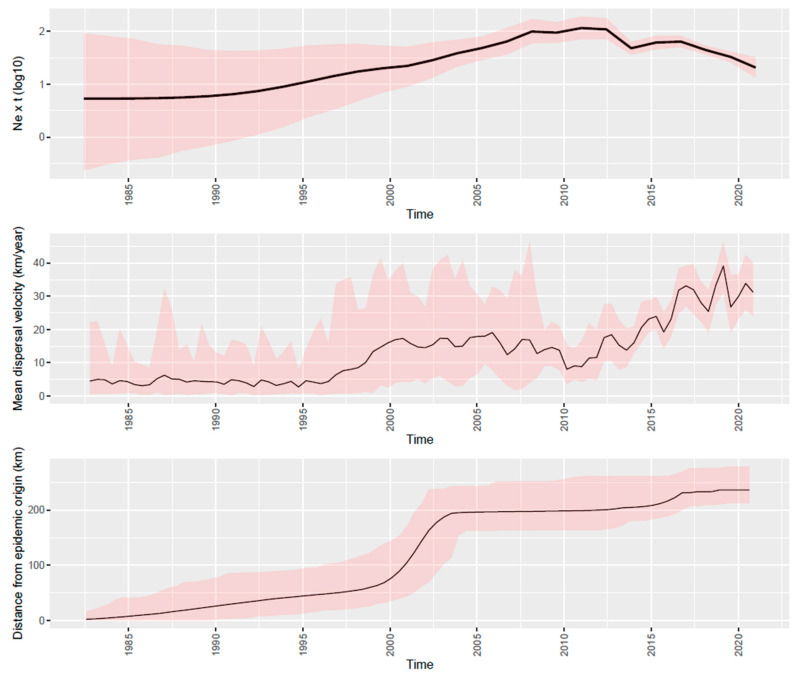
Depiction of relative genetic diversity (upper panel), mean dispersal velocity (middle panel), and distance from the epidemic origin (lower time) of PRRSV over time in Italy. Mean values are represented as a black line, while 95HPD intervals have been displayed as red-shaded areas.

The phylodynamic analysis revealed an evolutionary rate of 6.40 × 10^−3^ substitution/site/year (95HPD: 5.53∙10^−3^–7.36∙10^−3^ substitution/site/year). The introduction of the ancestor of the sampled strains was predicted approximatively in the ’80s. Overlapping results were obtained regardless of the implemented analysis (DTA, BASTA, or continuous phylogeography) (Figure S3). Thereafter, the viral population was featured by a progressive and relevant increase in relative genetic diversity (i.e., effective population size ∙ generation time; Ne × τ) until approximately 2010, when a marked decrease began and persisted until the end of the study, with a small rebound in 2015 (Figure 1).

### 3.3. Within Integrated Pig Production Chain Strain Flow

**Figure 2:**
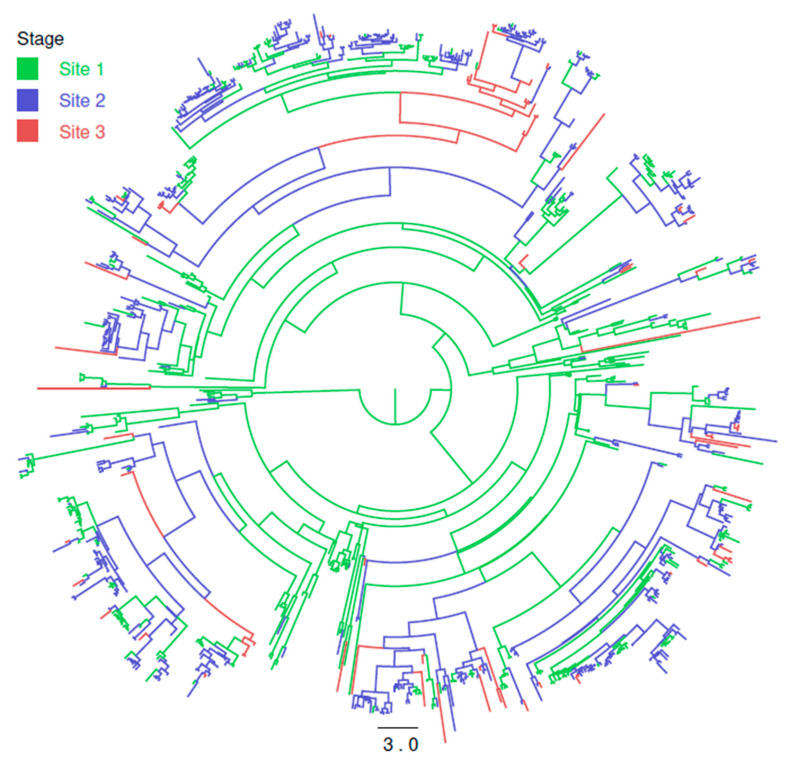
Maximum clade credibility tree of Italian PRRSV strains. Productive sites where the virus ancestors were estimated to circulate have been color-coded. The branch length is scaled in time (years).

The DTA, investigating the PRRSV strain migration among the different stages of pig production, demonstrates the directionality of the viral flux. The asymmetric migration model was preferred over the symmetric one (Bayesian Factor (BF) > 10), and the estimated migration rates supported the directionality from site 1 to site 2 to site 3. Moreover, the migration rate from sow herds to nurseries and from nurseries to finishing units were three and two times higher than the ones from finishing to sows, from finishing to nurseries and from sows to finishing. The viral flux from nurseries to sows units was comparable to the one from sows to nurseries. Therefore, the viral flux essentially followed the pig one, although with some exceptions (Figure 2).

### 3.4. Strain Migration among Integrated Pig Production Chains

**Figure 3:**
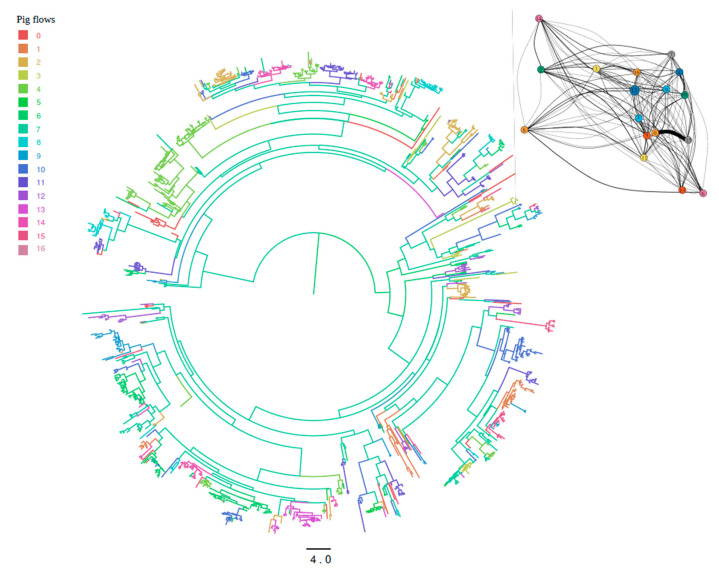
Maximum clade credibility tree of Italian PRRSV strains. Integrated pig flows (reported as numbers in the legend) where the virus ancestors were estimated to circulate have been color-coded. The branch length is scaled in time (years). Insert: Statistically supported migrations between integrated pig flows; the size of the arrow is proportional to the inferred migration rate.

The analysis of viral migration among integrated pig companies highlighted substantial compartmentalization among productive units since clades of the MCC tree demonstrated a clear tendency to include strains sampled from farms of the same integrated productive flow (Figure 3). Although rare, some among-company migration events were also present, which were confirmed by the identification of different significant migration rates that, although relatively low in absolute value (~1 migration event/year), connected different groups (Figure 3). Additionally, a limited number of integrated production flows showed significantly higher values (Figure 3).

**Figure 4:**
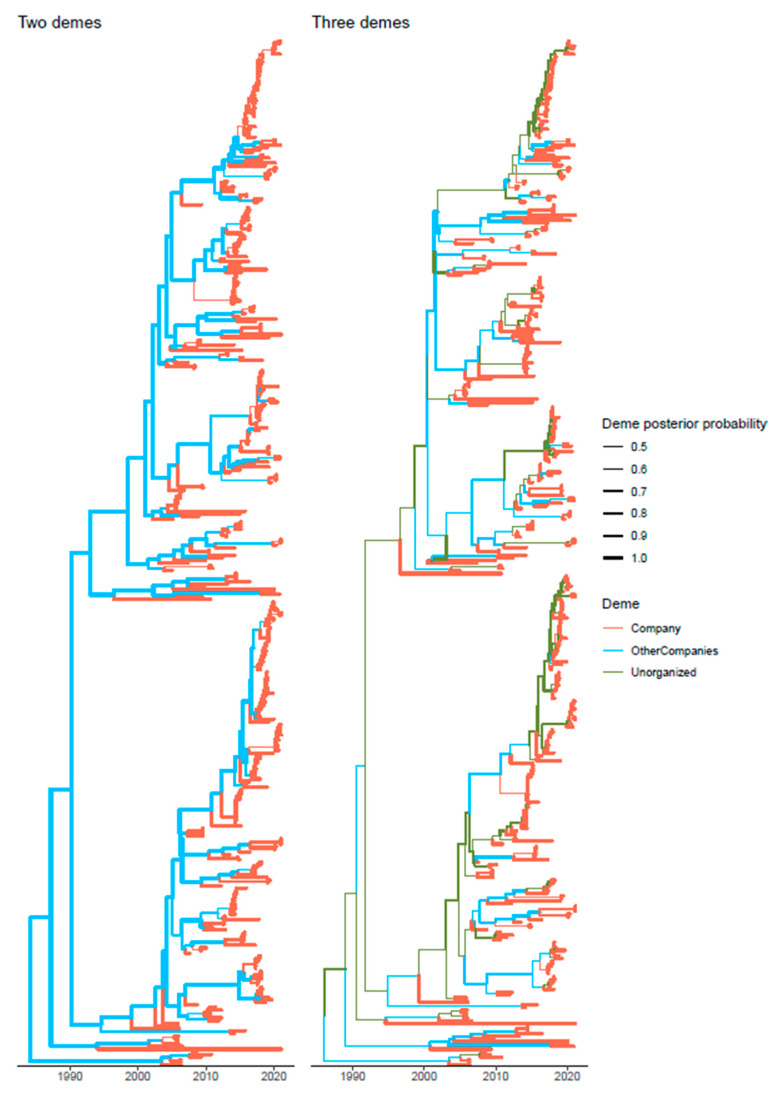
Structured coalescent-based phylogenetic tree of the samples included in the present study. Branch colors, as from legend, mark the inferred company where the ancestral strain was circulating, while branch width represents the posterior confidence of the inference. The trees reconstructed assuming just the Company and all other Italian pig production (left figure) and the one including also a third ghost deme (i.e., Company, other integrated companies, and unorganized/rural farms) (right figure) are reported.

The role of ghost demes, representative of unsampled integrated pig companies belonging to other commercial groups and to rural pigs was also investigated demonstrating its non-negligible impact in mediating the passage of PRRSV strains from one integrated pig flow to another (Figure 4). In the two demes model, the deme size of the ghost deme was predicted to be approximatively 40 times bigger than the *Company* one, while in the three demes model the size of the viral population in the other integrated companies and unorganized farms was ~4 and 60 times bigger than the *Company* one, respectively.

### 3.5. Continuous Phylogeography

**Figure 5:**
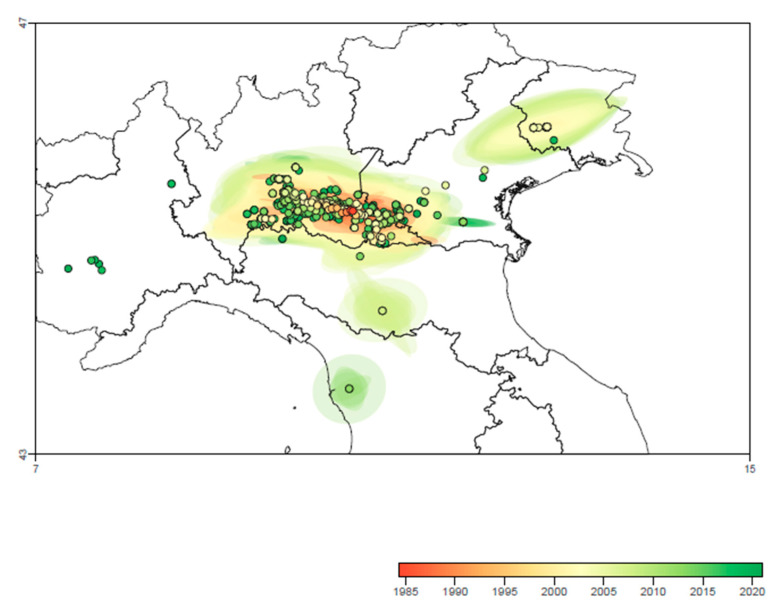
Reconstruction of spatio-temporal dispersal history of PRRSV in Central-Northern Italy based on the maximum clade credibility (MCC) trees and 80% HPD regions estimated through the continuous phylogeographic analysis. Nodes of the MCC tree are represented as circles colored according to their time of occurrence (see color-coded scale), and 80% HPD regions were computed for successive time layers and then superimposed using the same color scale reflecting time.

The continuous phylogeographic analysis suggested a most likely first introduction of PRRSV strain in the Regions of central part of Northern Italy (Lombardy, Emilia Romagna and Veneto), followed by a centrifuge spreading around the initial introduction site and thereafter to more peripherical areas in the East, such as Friuli Venezia Giulia (~2010), and West and Central Italy, such as Piedmont and Tuscany (~2015) (Figure 5).

The analysis of the wavefront distance (Figure 1) suggested a relatively slow increase of the involved areas during the first 2 decades of PRRSV circulation, which was followed by a more rapid expansion in the period 2000–2005, leading to the involvement of the final area distribution. Over the same period, the spreading speed progressively increased from the introduction to approximatively 1995, while a marked rise in the spreading velocity was observed until 2000. Finally, a new increase, although with minor fluctuations, featured the period from 2010 to the end of the study (Figure 1).

The effect of several environmental variables on PRRSV strain distribution was adequately statistically supported. Particularly, a lower tendency of PRRSV strain to be located in areas with higher values of altitude, road, and human population density was estimated. On the contrary, higher values of temperature annual range and seasonality, cropland usage, and pig density enhanced strain persistence in an area (BF > 10).

Similarly, some environmental variables, particularly road density, elevation, and mean annual temperature, proved to affect viral spreading speed (BF > 10). Road density and elevation acted as conductance (i.e., enhancing) factors, according to a least-cost diffusion model, while the annual mean temperature was classified as a resistance factor.

## 4. Discussion

PRRSV is probably the most frustrating infectious disease affecting commercial pig farming. Despite the development and application of several vaccines, no vaccination protocol has proven completely effective [13]. Great efforts have thus been devoted to improving biosecurity measures and pig management, with mixed fortunes. Particularly, attempts to reduce the area prevalence and between farms spreading were often unsuccessful and frequent breakages leading to new strain introduction occur [13,41].

The present study investigates PRRSV circulation in Italy and its drivers, largely confirming the limitations in PRRSV control despite the remarkable paid efforts. To this purpose, we obtained the broadest available ORF7 sequences dataset originating from the diagnostic activity of the largest Italian pig company in the period 2004–2021. The ORF7 was chosen for several reasons. The selection of a slightly more conserved region provided several advantages. It is well known that the variability of the ORF5 gene can pose challenges in PRRSV diagnosis and especially sequencing, creating a potential bias in the sequencing success based on primer affinity. This threat is largely reduced by selecting more stable regions as primer target, which guarantees a more representative sequence (i.e., strains) dataset. Additionally, unlike other ORFs, ORF7 was reported not to be prone to recombination [8,40], a phenomenon that, if not properly detected and accounted for, can severely bias the analysis and the results reliability. Nevertheless, unlike the common thought, the ORF7 is featured by a relevant genetic variability. The mean genetic distance of our dataset was 9%, with an interval from 0 to 18.8%. Accordingly, the formal test of phylogenetic signal (i.e., likelihood mapping) demonstrated that the strength of the phylogenetic information provided by the available sequence alignment was adequate to perform the analyses.

The origin of Italian PRRSV strains was predicted in the 1980s. Since the estimated PRRSV origin is far more ancient [42], it can be speculated that Italy remained PRRSV-free for a long time, potentially due to the structure of the swine industry and limited swine trade in the first half of the century. However, it must be kept in mind that the herein-estimated tMRCA refers to the currently circulating (and sampled) strains. Therefore, other lineages and their ancestors could have been circulating in Italy for a much longer time without being sampled due to being extinct or limited to certain companies, geographic areas, or production types (e.g., rural sector) that were not included in the study.

Despite these speculations on virus introduction, the following spreading pattern appears much clearer. After the initial introduction in the central part of Northern Italy, it centrifugally spread, affecting the high-density populated areas of Emilia Romagna, Lombardy and Veneto. Accordingly, the results of strain distribution demonstrated a positive association between pig density and PRRSV strain location, as well as the tendency of viruses to be present in an area with high cropland use and low human population and road density, which simply reflects the agricultural vocation of the affected areas. Similarly, studies performed in US demonstrated increments of swine density and being located in cultivated/managed areas to significantly increase the risk for PRRS outbreaks [43,44].

PRRSV diffusion appeared initially slow until an abrupt increase was observed around the middle of the 90s that led, with approximately a 5 years delay, to a significant expansion of the involved area reaching the final distribution range. Such rise in viral spreading closely mirrored the commercial expansion of the *Company* that, in the same years, acquired new farms located in different areas of Northern Italy. The increase in animal movements and the broader spanned distances can thus justify this finding. In fact, the observed progressive increase in spreading speed can be expected since the broader the affected area, the higher the number of infected farms and therefore the number of potential infection sources and contacts. More surprisingly, after the maximal territorial distribution was reached, the virus continued to move at a comparable or even higher rate. This evidence testifies an intense strain circulation within the considered area and suggests the limits of implemented biosecurity and control measures. Interestingly, in the same period, the introduction and following increase of a second Italian PRRSV clade (Clade B) was previously reported [10], progressively superseding the previous variant. Therefore, a higher viral virulence or decrease animal immune-protection cannot be excluded and could have contributed to the observed pattern. Of note, the viral population increased constantly from the tMRCA to 2010, when a decrease phase began. In the same years, the *Company* sanitary policy started focusing particularly on gilt acclimatization rather than on vaccination only. Particularly, a protocol based on a combination of vaccinations followed by forced infection with autochthonous field strains, achieved by exposing gilts to runts of the same farm, was applied on a large scale. Therefore, it can be speculated that such intensive immunization protocol effectively limited viral circulation in the first farming steps and, combined with following strict biosecurity measures, had a significant effect on viral circulation in the downstream productive chain.

Nevertheless, the overall picture suggests that even if local biosecurity and control measures were at least partially effective in decreasing viral prevalence, the circulation over long distances was not prevented, leading to the relatively frequent introduction of new strains in distantly related farms. The hierarchical, vertical *Company* organization can largely explain the observed scenario; a single farrowing unit provides pigs to several weaners located in different Italian areas and/or regions that, in turn, supply pigs to several fattening units. Therefore, according to this picture, PRRSV appears to follow a spreading pattern mainly driven by pig flow, similar to what previously observed by Pesente et al. (2006), who reported the movements of carrier animals from sow herds to nursery and from nursery to finishing as the main route of infection in an integrated product system [45]. Similar evidence was recently demonstrated in North American farming [19]. Other studies have previously reported that animal movement represents one of the most important risk factors for the spreading of PRRSV and other infections in food-producing animals [19,46,47,48]. DTA analysis supports this hypothesis since an asymmetric PRRSV migration model among productive stages was preferred over the symmetric one and more intense connections from sow herds to nurseries and from nurseries to finishing units were predicted. Nevertheless, a non-negligible flux of PRRSV dissemination from nurseries to sow farms was also noticed, confirming the importance of downstream farms as sources of the virus [44].

While this result can be taken for granted and easily predicted [19], it is more challenging to evaluate how the overall system was able to prevent viral spreading among integrated pig production units, creating effective compartments. The phylogenetic tree evaluation clearly showed the presence of clusters, including strains collected from single integrated pig farms, demonstrating the effect of vertical integration as a control strategy. Still, the occurrence of several exceptions and the presence of different migration rates among those companies (estimated using the structured coalescent) testify to the occurrence of several breakages, mirroring what was observed in US farming system [44] and reinforcing the importance of PRRSV collateral spread.

A similar study performed in Italy on Avian coronavirus suggested much stronger compartmentalization in chicken farming [49]. The features (e.g., different life span, more frequent interaction with veterinarians and technicians, and need for a higher number of travels between birth and slaughtering) of the two productive systems (pigs vs. chickens) surely played a role. However, it must be also stressed that in the previous study different commercial companies were compared and considered as separate demes. Contacts between companies with different owners can be considered limited. On the contrary, in the present study, all considered farms were part of the same *Company*, although divided into integrated, vertical pig flows. A partial sharing and overlapping of the facilities and services (veterinarians, technicians, trucks, etc.) occurs, thus increasing the risk of virus introduction through animal movements and/or contaminated fomites. Accordingly, road density was proven to be significantly associated with viral migration speed. Therefore, the relevance of indirect contacts among distant farms and production chains, in addition to pig movements demonstrate in the present study and Makau et al. (2021) [19], was thus proven pivotal. The role of indirect contacts on effective PRRSV transmission has been amply demonstrated and represents one of the main challenges in infection control and, especially, eradication [11,41,50].

Interestingly, while the animal density was the only factor with a positive effect on viral spreading speed for poultry, it was not significant for PRRSV in Italy. PRRSV has been detected and isolated in aerosols at relatively high distances, thus highlighting the relevance of bioaerosols in control programs [50]. However, in the present study, although pig density was positively correlated to strain presence and persistence in an area, it did not affect viral spreading speed. Aerial spreading seems therefore to play at least a secondary role in PRRSV introduction into new farms. These pieces of evidence are in contrast with what was previously reported by Pitkin et al. (2009) [51]. Whether this is due to the features of Italian pig farms (average herd size, farm structure, aeration, etc.), environment, and/or other local peculiarities, and the extent to which such conclusion can be generalized to other contexts, will need further investigation.

Of note, similar to what was demonstrated for the poultry industry, unsampled companies largely represented by less organized companies or single semi-rural or rural farms (i.e., the included ghost demes) appear to play a significant role in mediating the contact among different units of the considered integrated company [49]. Additionally, for pig farming, PRRSV dissemination was largely featured by transmission between non-commercially-related farms rather than within a production systems [44].

The structured coalescent analysis predicted several intermediated branches (i.e., ancestral unsampled strains), linking groups of viruses collected in the *Company*, that circulated in other groups and/or farms. Likely, the lower economic, managerial, and logistic resources of those farms can justify a higher viral presence and thus “infectiveness” for other farms.

The predicted deme population size was significantly lower in the *Company* compared to the “ghost” deme, which did not reflect the actual proportion of raised pigs, since about 10–15% of Italian pig production only is held by the *Company*. Even more interestingly, when a third deme was introduced in the model representing other integrated pig companies, the respective deme sizes of the *Company* and other Italian integrated groups were essentially proportional to the number of raised animals, while the third deme, i.e., small-independent farms and rurally raised pigs, still showed a disproportionally large population. This scenario could thus substantiate the higher capability of integrated companies in reducing PRRSV presence and prevalence through effective and systematic control measures application.

While these estimations are surely featured by a relevant uncertainness and the prediction of the behavior of demes for which no sequences are available might seem unreliable, such a modelling approach has been proven effective in depicting the epidemiology of other veterinary and human infectious diseases [24,26,27]. Therefore, even if specific links among farms and companies must be interpreted with caution, the overall pattern should be considered for a better organization of future control plans.

If effective control at the regional level must be achieved, a shared control approach that simultaneously targets multiple farms within a relatively large region, based on strong communication and collaboration among productive sites and operators (owners), must be pursued to minimize the risk of re-introduction of the virus into any single farm, as previously suggested [13,52]. Unfortunately, although diagnostic and sequencing activity can be considered intensive in Italy, epidemiological data and sequence obtained by different, independent laboratories are rarely shared or made publicly available, hindering the understanding of PRRSV behavior, epidemiological links, and contributions of swine farming actors. Particularly, the present study results are based on high-level integrated pig company located in Northern Italy. While this can be considered representative of other intensive productive systems, as demonstrated by the ghost deme estimation performed through structured coalescent analysis, the results can hardly be confidently extended to rural farms, especially those located in Central and Southern Italy where the productive system is radically different. Therefore, additional studies and the sharing of PRRSV sequences information would be necessary to properly investigate this context and complete the pictured of the Italian PRRSV epidemiology.

Finally, environmental and climatic variables also seem to affect PRRSV presence and spreading. Particularly, low temperatures and high climatic variability (temperature annual range and seasonality) were positively associated with viral migration and persistence, likely because these conditions indirectly act on host susceptibility and viral persistence in the environment and fomites. These results confirm previous ones, suggesting the spread of PRRSV from a swine production site to another affected by lower temperature, relative humidity, and sunlight intensity [51,53,54]. Similarly, when the PRRSV-free status persistence after the establishment of a new site was assessed, non-winter months were associated with longer maintenance of the status [41].

More surprisingly, elevation also appeared to positively affect PRRSV spreading speed, a piece of evidence that could seem counterintuitive. Most of the farrowing units are sited in elevated areas, a position that typically guarantees higher isolation and distance from other pig farms. In fact, a lower tendency of PRRSV strain to be located in areas with high altitude values was proven in the present study and also in the US [43,48], enforcing the effectiveness of such measures. However, considering that the pig and thus PRRSV strain flows originate from these units, the reconstructed migration path will often pass through elevated areas. Therefore, the correlation between the viral spreading flow and elevated position of the farm of origin can explain the “artefactual” enhancing role of altitude on the viral spread and, at the same time, the lower tendency of the virus to be present in such areas.

## 5. Conclusions

Overall, the present study testifies to the contribution of several factors in shaping PRRSV epidemiology, at least in Italy. Animal management, and especially proper gilt acclimatization (i.e., immunization) and independence of pig flows (i.e., biosecurity), were demonstrated to be highly effective in limiting viral spread and thus constraining viral population size. A clear advantage in preventing and limiting viral circulation of highly organized companies over small-rural one appeared evident. On the contrary, small-scale production represents a threat for major ones, likely incrementing the local farm density in presence of ineffective control and biosecurity measures, which determines a higher infectious pressure. Despite all paid efforts, viral transmission could not be prevented in high-management integrated companies either and different environmental factors were proven relevant risk factors for viral persistence and spreading. Of note, the herein-reported results are largely in agreement with the evidence obtained from other studies performed in the US [17,19,20]. Besides strengthening the robustness of the obtained results, the prominent role of modern, intensive pig farming organization in affecting PRRSV epidemiology, rather than the local environment and managerial peculiarities, is supported.

Based on such an intricate and multifactorial net of PRRSV epidemiology determinants, the infection can be effectively controlled through comparable multi-level intervention strategies, including proper planning of farm structure and location, adequate animal management and immunization within farms, the preservation of independent pig flows, and strict cooperation and information exchange among different companies and operators working in the same area [52].

## Data Availability

Due to privacy reason imposed by the Company policy, sequences are not publicly available. Data can be requested to the corresponding author.

## Supplementary Materials

The following are available online at https://www.mdpi.com/article/10.3390/v13122510/s1, Figure S1: Rasters of environmental variables used in the present study. Figure S2: ORF7-based phylogenetic tree depicting the distribution of the sequences obtained in the present study (colored in red) on the background of other Italian (colored in blue) and international (colored in black) sequences. PRRSV type II sequences were selected as the outgroup to root the phylogenetic tree. Figure S3: Density plot of the evolutionary rate (upper figure) and tMRCA (lower figure) posterior probability estimated using different methods (color-coded).
