# Supplementary figures and images for: Porcine Reproductive and Respiratory Syndrome (PRRS) Epidemiology in an Integrated Pig Company of Northern Italy: A Multilevel Threat Requiring Multilevel Interventions
Source: Viruses. 2021 Dec 14;13(12):2510. doi: 10.3390/v13122510 (PMC8705972; doi:10.3390/v13122510)

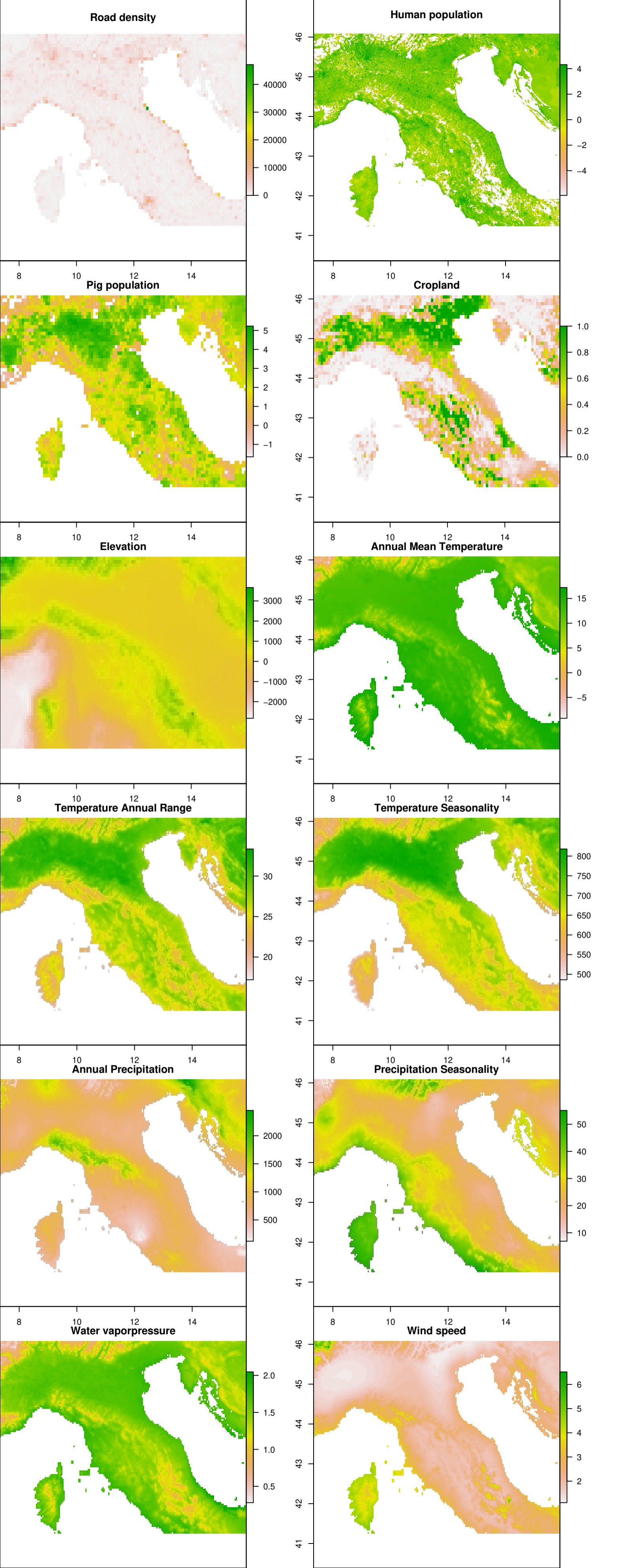

Supplement: Supplementary file 1 [file viruses-13-02510-s001.zip › Figure S1.pdf]

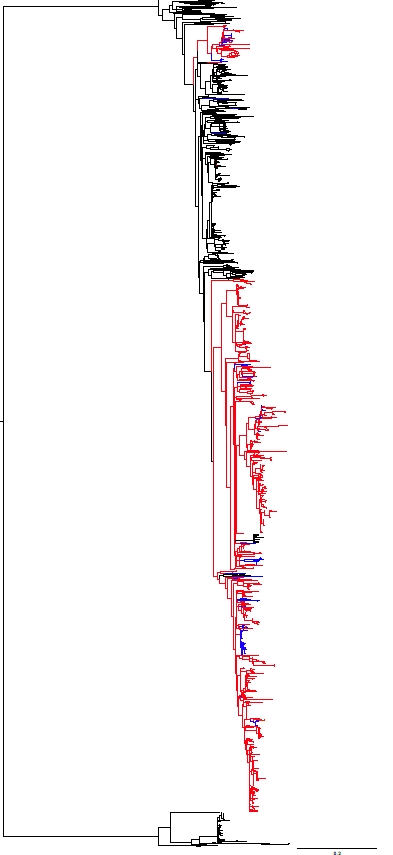

Supplement: Supplementary file 1 [file viruses-13-02510-s001.zip › Figure S2.png]

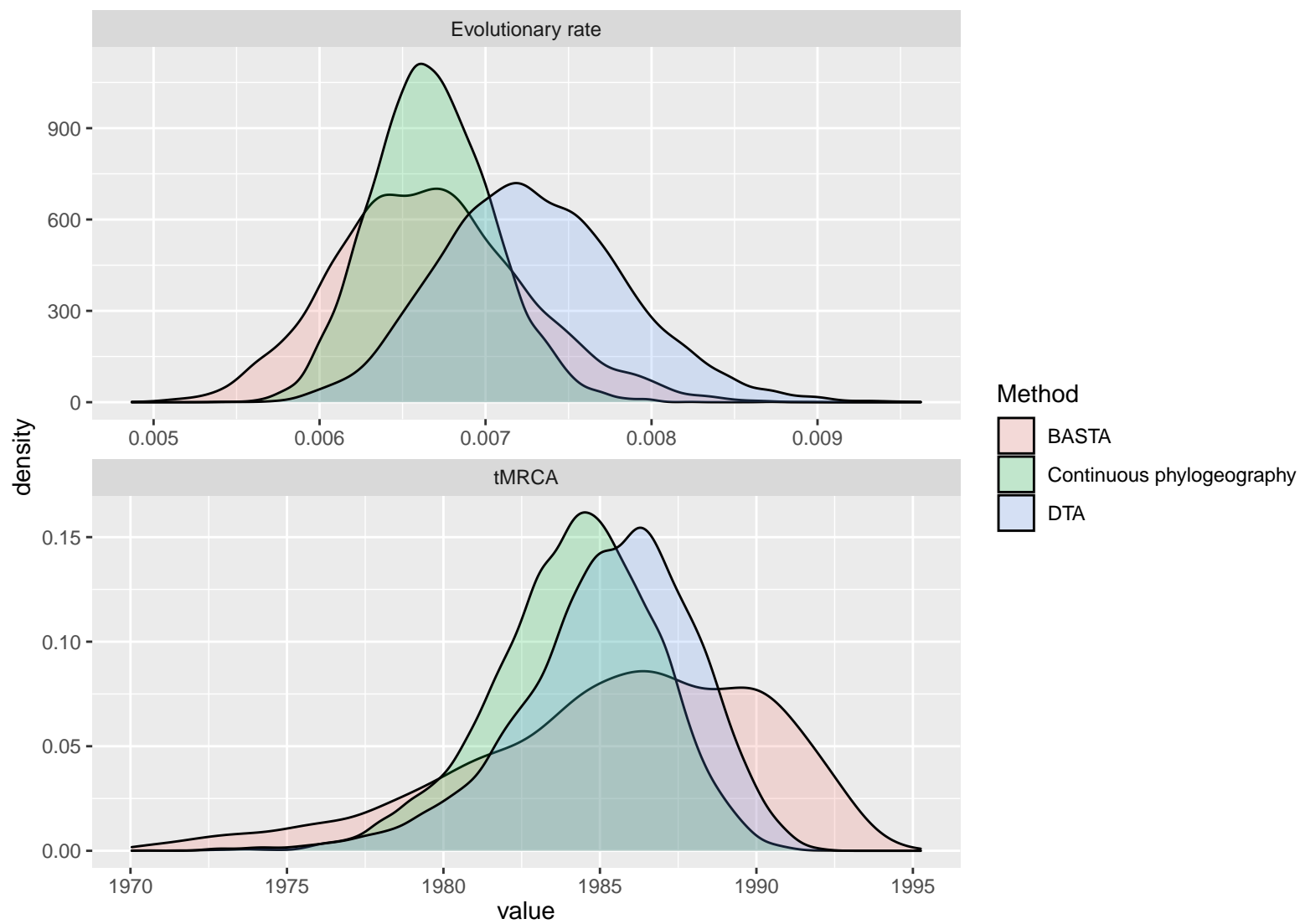

Supplement: Supplementary file 1 [file viruses-13-02510-s001.zip › Figure S3.pdf]
